# Alcohol Withdrawal Rates in Hospitalized Patients During the COVID-19 Pandemic

**DOI:** 10.1001/jamanetworkopen.2021.0422

**Published:** 2021-03-03

**Authors:** Ram A. Sharma, Keshab Subedi, Bayo M. Gbadebo, Beverly Wilson, Claudine Jurkovitz, Terry Horton

**Affiliations:** 1 Department of Psychiatry, Christiana Care, Newark, Delaware; 2 Value Institute, Christiana Care, Newark, Delaware

## Abstract

This cohort study examines whether alcohol withdrawal rates among hospitalized patients with alcohol use disorder increased during the coronavirus disease 2019 (COVID-19) pandemic.

## Introduction

Coronavirus disease 2019 (COVID-19) is disrupting communities across the globe, causing physical, mental, and financial distress.^1^ Economic crises have been associated with increased alcohol consumption.^2^ Necessary public health measures may exacerbate isolation and stress, negatively impacting those who are at risk for harmful alcohol use. Increased alcohol use has been documented in the US and other countries during the pandemic, and a recent study^3^ has identified associated consequences. Alcohol withdrawal (AW) is a potentially dangerous complication of alcohol use disorder (AUD) in up to 8% of all hospitalized patients with AUD.^4^ AW has been suspected to worsen after the COVID-19 stay-at-home orders,^5^ but, to our knowledge, no objective data have been reported in the literature. We hypothesized that AW rates in hospitalized patients with AUD increased during the pandemic and conducted a cohort study at Christiana Care, a large, tertiary care hospital system in Newark, Delaware.

## Methods

After receiving institutional review board approval from Christiana Care, we extracted admission and demographic information from the electronic health records data warehouse for all patients hospitalized between January 1, 2018, and September 22, 2020. A limited data set as per institutional review board definition was used. Informed consent was waived by the Christiana Care institutional review board in accordance with the Office for Human Research Protections regulations 45 CFR 46.116(d). The Strengthening the Reporting of Observational Studies in Epidemiology (STROBE) reporting guidelines relevant to our study were followed.

We used a revised Clinical Institute Withdrawal Assessment for Alcohol^6^ score of 8 or higher to identify AW in hospitalized patients. Summary statistics were calculated for unique patients in 3 periods in 2020: before (January 1 to March 24), during (March 25 to May 31), and after (June 1 to September 22) the statewide stay-at-home period. Incidence rates for AW were computed for biweekly periods in 2018, 2019, and 2020. Furthermore, incidence rate ratios (IRRs) and 95% CIs were calculated for each period in 2020 using the same periods in 2019 and mean of 2018 and 2019 as reference, to account for seasonal variations. Significance was set at 2-tailed *P* < .05. SAS statistical software version 9.4 (SAS Institute) was used for all calculations. Data analysis was performed from October to December 2020.

## Results

The study population included 340 patients (overall mean [SD] age, 52.3 [14.2] years) who received a diagnosis of AW before the stay-at-home order (mean [SD] age, 52.6 [14.2] years; 101 women [29.7%]; 73 Black patients [21.5%]; 18 Hispanic patients [5.3%]), 231 patients who received a diagnosis during the stay-at-home period (mean [SD] age, 52.3 [14.8] years; 74 women [32.0%]; 44 Black patients [19.1%]; 12 Hispanic patients [5.2%]), and 507 patients who received a diagnosis after the stay-at-home period (mean [SD] age, 52.2 [13.4] years; 156 women [30.8%]; 114 Black patients [22.5%]; 25 Hispanic patients [4.9%]). Patient characteristics were similar among the 3 periods. The rate of AW in hospitalized patients was consistently higher in 2020 compared with both 2019 and the average of 2018 and 2019, although the difference was larger in the period after the stay-at-home order (Figure). The largest IRR in 2020 vs 2019 (IRR, 1.84; 95% CI, 1.30-2.60) occurred in the last 2 weeks of the stay-at-home order (Table). AW rates in hospitalized patients increased by 34% in 2020 during the pandemic (March 25 to September 22) compared with the same period in 2019 (IRR, 1.34; 95% CI, 1.22-1.48).

**Figure.**
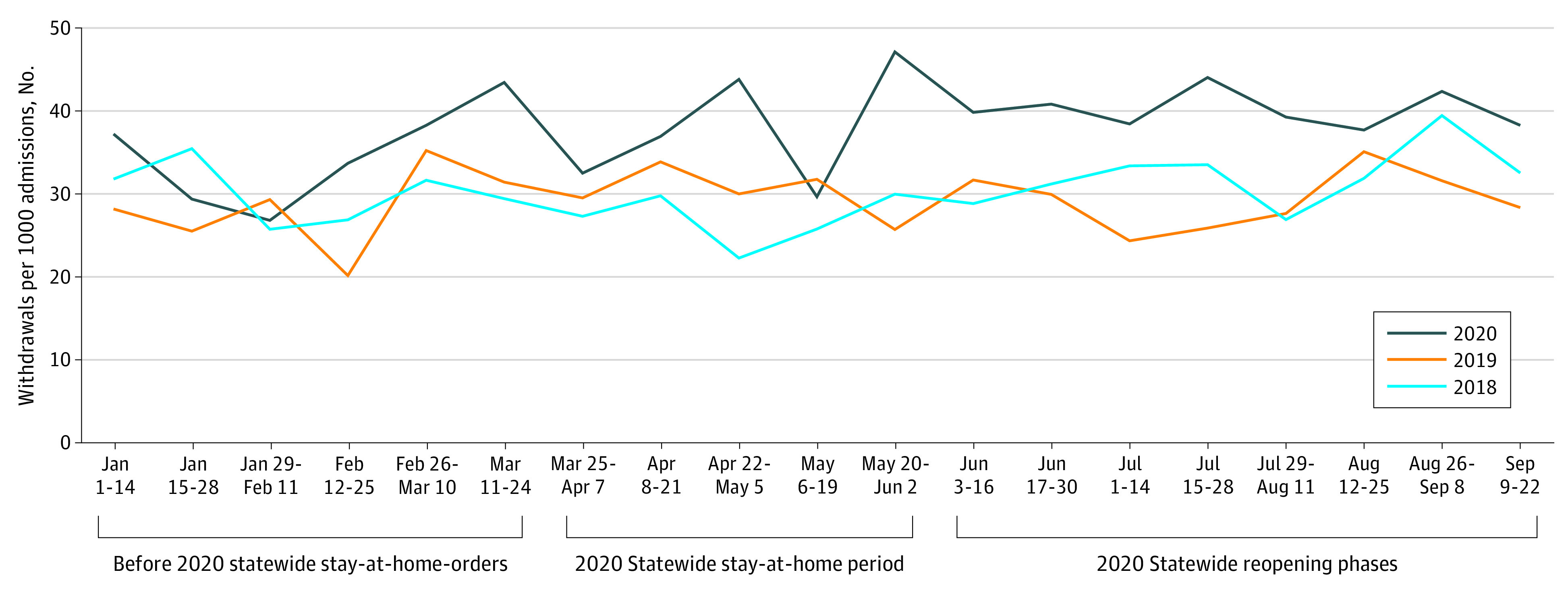
Trend of Alcohol Withdrawal in Hospitalized Patients, per 1000 Weekly Admissions, From January 1 to September 22 in 2018, 2019, and 2020 Alcohol withdrawal was defined as a revised Clinical Institute Withdrawal Assessment for Alcohol score greater than or equal to 8.

**Table.** Incidence Rate of AW Among Hospitalized Patients, for Biweekly Periods Between January 1 and September 22 in 2018, 2019, and 2020^a^

Biweekly period^b^	Cases of AW in hospitalized patients, No.			IR, AW cases/1000 admissions			IRR (95% CI) for 2020	
	2018	2019	2020	2018	2019	2020	2019 IR used as reference	Mean of 2018 and 2019 IRs used as reference
January 1-14	62	58	75	31.99	28.35	37.46	1.32 (0.94-1.86)	1.24 (0.93-1.65)
January 15-28	69	51	57	35.70	25.64	29.55	1.15 (0.79-1.68)	0.97 (0.71-1.33)
January 29 to February 11	50	59	53	25.88	29.50	26.96	0.91 (0.63-1.32)	0.97 (0.7-1.35)
February 12-25	53	40	66	27.03	20.24	33.92	1.68 (1.13-2.49)	1.44 (1.05-1.97)
February 26 to March 10	60	73	78	31.85	35.45	38.52	1.09 (0.79-1.50)	1.14 (0.86-1.51)
March 11-24	57	66	68	29.61	31.62	43.73	1.38 (0.98-1.94)	1.43 (1.06-1.92)
March 25 to April 7	53	59	43	27.46	29.68	32.70	1.10 (0.74-1.63)	1.14 (0.8-1.62)
April 8-21	59	65	51	29.93	34.08	37.17	1.09 (0.76-1.57)	1.16 (0.84-1.61)
April 22 to May 5	43	60	69	22.37	30.18	44.12	1.46 (1.03-2.06)	1.67 (1.23-2.27)
May 6-19	50	64	47	25.91	31.95	29.82	0.93 (0.64-1.36)	1.03 (0.73-1.45)
May 20 to June 2	58	52	82	30.16	25.86	47.45	1.84 (1.30-2.60)	1.7 (1.28-2.26)
June 3-6	55	63	72	28.99	31.88	40.09	1.26 (0.90-1.77)	1.32 (0.98-1.77)
June 17-30	58	61	74	31.37	30.12	41.11	1.36 (0.97-1.91)	1.34 (1.0-1.79)
July 1-14	64	48	69	33.60	24.48	38.70	1.58 (1.09-2.28)	1.34 (0.99-1.81)
July 15-28	61	52	80	33.74	26.01	44.35	1.70 (1.20-2.41)	1.49 (1.12-1.98)
July 29 to August 11	52	55	71	27.06	27.81	39.53	1.42 (1.00-2.02)	1.44 (1.07-1.94)
August 12-25	59	65	72	32.08	35.31	37.95	1.07 (0.77-1.50)	1.13 (0.85-1.51)
August 26 to September 8	77	59	81	39.71	31.77	42.65	1.34 (0.96-1.87)	1.19 (0.9-1.57)
September 9-22	64	57	69	32.72	28.50	38.53	1.35 (0.95 1.92)	1.26 (0.94-1.69)

Abbreviations: AW, alcohol withdrawal; IR, incidence rate; IRR, incidence rate ratio.

^a^ AW was defined as a revised Clinical Institute Withdrawal Assessment for Alcohol score greater than or equal to 8.

^b^ January 1 to March 24 was the pre–statewide stay-at-home order period. March 25 to May 31 was the statewide stay-at-home period. June 1 to September 22 was the statewide reopening phase.

## Discussion

The association of the COVID-19 pandemic with AUD and AW has been much debated.^5^ This cohort study found an overall increase in AW rates in 2020, with a peak at the end of the stay-at-home order. Moreover, increased AW rates continued during the reopening phases. It is not clear why IRRs were higher in 2020 before the stay-at-home orders. Although the use of the revised Clinical Institute Withdrawal Assessment for Alcohol to identify AW limits the false-positive rate, it may underestimate the true AW rate and may be a limitation of the study. These findings suggest negative associations of the pandemic with AW.

Stress, anxiety, disrupted treatment plans, and increased alcohol use might be factors associated with higher rates of AW, because higher rates persisted during the reopening phases. With the recent surge in COVID-19 cases, many states might revert to stay-at-home orders and this trend may worsen. Increased vigilance to identify AW among hospitalized patients and to use systematic screening will be pivotal in the management of AW.
